# A phone and text message intervention to improve physical activity in midlife: initial feasibility testing

**DOI:** 10.1080/21642850.2022.2049796

**Published:** 2022-03-10

**Authors:** Jeff C. Huffman, Lauren E. Harnedy, Christina N. Massey, Alba Carrillo, Emily H. Feig, Wei-Jean Chung, Christopher M. Celano

**Affiliations:** a Harvard Medical School, Boston, MA, USA; b Department of Psychiatry, Massachusetts General Hospital, Boston, MA, USA; c Instituto Polibienestar, University of Valencia, Valencia, Spain

**Keywords:** Mid-life, motivational interviewing, physical activity, positive psychology, text message intervention, well-being

## Abstract

**Background:**

Physical activity during midlife (ages 45-64) plays a major role in the prevention of chronic and serious medical conditions. Unfortunately, many midlife adults struggle to be physically active in the setting of low levels of psychological well-being and the management of multiple confluent sources of stress. Effective, scalable, midlife-specific interventions are needed to promote physical activity and prevent the development of chronic medical conditions.

**Objectives:**

In an initial proof-of-concept trial, we assessed the feasibility and acceptability of a new, midlife-adapted, phone- and text message-based intervention using positive psychology (PP) skill-building and motivational interviewing (MI) techniques. We secondarily analyzed post-intervention changes in accelerometer-measured physical activity and self-reported outcomes.

**Methods:**

The PP-MI intervention included six weekly phone sessions with a study trainer, with completion of PP activities and physical activity goals between calls, and in the subsequent six weeks briefer phone check-ins were conducted. Text messages over the 12-week intervention period were utilized to support participants and identify barriers to goal completion. Feasibility (session completion rates) and acceptability (participant ratings of intervention ease and utility) were assessed via descriptive statistics, and pre-post improvements in psychological, functional, and physical activity outcomes at 12 weeks were examined via mixed effects regression models.

**Results:**

Twelve midlife adults with low baseline physical activity enrolled in the single-arm trial. Overall, 76.8% of all possible sessions were completed by participants, and mean ratings of weekly phone sessions were 8.9/10 (SD 1.6), exceeding our *a priori* thresholds for feasibility and acceptability. Participants demonstrated generally medium to large effect size magnitude improvements in accelerometer-measured physical activity, psychological outcomes, and function.

**Conclusions:**

A novel, midlife-specific phone- and text-based PP-MI intervention was feasible and had promising effects on physical activity and other clinically relevant outcomes, supporting next-step testing of this program via a randomized controlled trial.

Midlife adults (age 45-64) represent over 25% of Americans and are the most rapidly growing age group in the United States (U.S. Census Bureau, 2010). Midlife is a crucial time period for the health of individuals, with many chronic conditions arising during this period, limiting function and leading to risk of additional serious conditions. For example, type 2 diabetes (Centers for Disease Control and Prevention, n.d) and hypertension (Fryer, Ostchega, Hales, Zhang, & Kruszon-Moran, 2017) most commonly arise during midlife, with the subsequent onset of heart disease immediately following this life stage (Virani et al., 2021).

Physical activity during midlife is associated with better overall health, lower rates of heart disease, and reduced mortality (Kodama et al., 2013). Despite physical activity’s critical role in health, many midlife adults are unable to initiate or maintain recommended levels of physical activity. Indeed, despite the clear importance of physical activity in promoting health and survival, fewer than 20% of midlife persons regularly engage in adequate physical activity (Tucker, Welk, & Beyler, 2011).

There likely are several reasons for low engagement in physical activity in midlife. One important contributor to low physical activity during this life phase is daily life stress, which can interfere with self-care efforts (Lachman, Teshale, & Agrigoroaei, 2015). Many midlife adults experience competing demands related to greater job responsibilities, caregiving of (younger and older) family members, and financial stress (Lachman et al., 2015). These elements can combine to greatly increase daily life stress and limit their ability to manage their health (Lachman et al., 2015; Madva et al., 2018). Furthermore, overall levels of positive psychological well-being appear to reach a nadir in midlife (Blanchflower & Oswald, 2008). Finally, many midlife adults experience substantial time pressure that can impede physical activity performance.

Motivational interviewing (MI), a counseling strategy designed to resolve ambivalence and enhance motivation, (Miller & Rollnick, 2012) is a promising option to promote physical activity in midlife persons. MI-based programs can be delivered remotely, which may increase their reach. However, MI-alone interventions have only led to small improvements in health behavior adherence in medical populations (standardized mean difference = 0.19; O'Halloran et al., 2014) suggesting that without additional components this approach may be insufficient to improve longitudinal health and prevent the onset of additional medical conditions in midlife.

Psychological well-being also may play an important role in physical activity and overall health in midlife. Positive psychological well-being is prospectively linked to increased physical activity and improved health outcomes, including reduced rates of heart disease and lower overall mortality, independent of sociodemographic and medical factors (DuBois et al., 2015; Levine et al., 2021). Positive psychology (PP) interventions, which involve the cultivation of positive psychological constructs through deliberate and systematic exercises (e.g. acts of kindness, using personal strengths, recalling positive life events), Seligman, Steen, Park, & Peterson, 2005 consistently improve well-being (Bolier et al., 2013; Carr et al., 2021; Sin & Lyubomirsky, 2009). There have been few studies of PP-based interventions designed to boost well-being and physical activity, but PP-based interventions have led to increased physical activity in medical settings in such projects, (Huffman et al., 2020; Peterson et al., 2012) potentially mediated through better mood, greater motivation and energy, increased perceived social support, and increased self-efficacy (Huffman, DuBois, Millstein, Celano, & Wexler, 2015a; Zambrano et al., 2020a).

Combining PP and MI could have powerful effects on physical activity in midlife persons (see theoretical model Huffman et al., 2015a, Figure 1). The MI component could increase physical activity by resolving ambivalence, enhancing motivation, increasing internal locus of control, and enhancing self-efficacy for exercise (Hawkins, 2010; Hettema, Steele, & Miller, 2005; Lundahl et al., 2013). Furthermore, the PP component could boost positive affect and reduce depression, both of which are linked to greater physical activity (Levine et al., 2021; Zambrano et al., 2020a). In addition, PP content could increase engagement in MI. PP interventions increase optimism, outcome expectancy, and self-efficacy, (Lee, Robin Cohen, Edgar, Laizner, & Gagnon, 2006; Lyubomirsky & Layous, 2013; Meevissen, Peters, & Alberts, 2011) along with perceived social support (Fredrickson, 2001; Majer, Jason, & Olson, 2004). These factors have been linked to greater effectiveness of MI and other behavioral interventions (Goossens, Vlaeyen, Hidding, Kole-Snijders, & Evers, 2005; Scheier et al., 2007). Finally, midlife-specific stress reduction content added to the MI-based health behavior change program could further promote health by directly addressing sources of stress identified by midlife persons as factors that can lead to lesser participation in health behaviors (Madva et al., 2018). We chose this intervention, as opposed to other options, given that it specifically addresses numerous factors (low motivation, low well-being, numerous sources of stress) that appear to impede midlife physical activity based on our literature review and developmental work outlined below.

**Figure 1.**
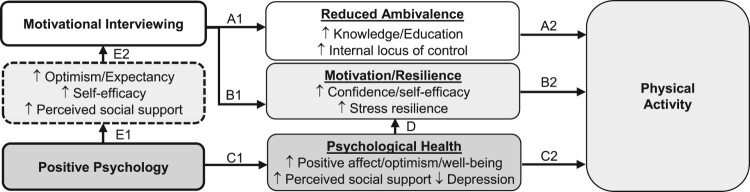
Theoretical model outlining potential mechanisms of intervention effects.

To be effective, such a physical activity program for midlife adults would need to be efficient and accessible, and it would need to address the barriers to physical activity commonly experienced by this population. Remotely delivered interventions reduce the time, financial, and transportation burdens of in-person programs, and both phone- and text message-based programs have led to improvements in physical activity, (Huffman et al., 2020; Huffman et al., 2021b) with text messaging programs for physical activity having a small-to-medium effect size (Cohen’s *d *= .31-.38 Smith, Duque, Huffman, Healy, & Celano, 2020), though the evidence for their impact is mixed (Nguyen, Gill, Wolpin, Steele, & Benditt, 2009; Smith et al., 2020; Wang et al., 2015). Additionally, text messages for physical activity appear to work best as part of multicomponent programs, rather than alone, (Smith et al., 2020) and they can be used to reinforce principles that were previously introduced in more detail by phone or in writing.

Despite the importance of promoting physical activity and well-being in midlife to improve health, assist in the management of existing medical conditions, and prevent cardiovascular disease, there has been very limited study of interventions that are tailored to midlife persons or address specific barriers to self-management (e.g. time pressure, caregiving burden) common in this population. Furthermore, few programs have focused on the promotion of psychological well-being in midlife, despite the fact that PP exercises are simple to deliver and consistently enhance well-being constructs.

Accordingly, using information from qualitative interviews and a small phone-based pilot study in midlife persons, (Huffman et al., 2021b; Madva et al., 2018) we developed a midlife-adapted, phone- and text-based intervention to promote physical activity in inactive midlife persons. The intervention combines elements of PP with MI and midlife-specific stress reduction content. We then tested this 12-week program in a single-arm proof-of-concept trial to examine its feasibility, acceptability, and potential impact. We hypothesized that the intervention would surpass *a priori* benchmarks for feasibility and acceptability, and would show small-to-medium effect size magnitude improvements in psychological outcomes and accelerometer-measured physical activity.

## Methods

**Overview.** This was a single-arm proof-of-concept trial examining the feasibility and preliminary impact of a PP-MI intervention, delivered via phone and text messages, among inactive midlife adults enrolled from the primary care practices affiliated with an urban academic medical center. Participants were enrolled between September and October 2020, and all participants provided informed consent. The study was approved by the health center’s institutional review board prior to study initiation (IRB approval: 2020P001884). We aimed to enroll between 10–12 participants over a two-month period given the availability of research staff for this unfunded feasibility trial and our use of similar samples when examining initial proof of concept of behavioral interventions prior to next-step testing (Celano et al., 2019; Celano, Freedman, Beale, Gomez-Bernal, & Huffman, 2018; Huffman et al., 2021b).

**Intervention development.** The PP-MI intervention was developed utilizing the NIH Stage model's conceptual framework of intervention development (Onken, Carroll, Shoham, Cuthbert, & Riddle, 2014). Our prior developmental work (Gomez-Bernal et al., 2019; Madva et al., 2018) had been Stage IA along this framework, with both a prior proof-of-concept trial (Huffman et al., 2021b) and the current trial representing Stage IB intervention trials. More specifically, our prior work included reviewing prior literature on midlife interventions, (Gomez-Bernal et al., 2019) conducting qualitative assessments examining midlife stressors and health behaviors, (Madva et al., 2018) reviewing the literature on PP-based interventions, (Carr et al., 2021; Cohn, Pietrucha, Saslow, Hult, & Moskowitz, 2014; Moskowitz et al., 2012) conducting analyses of prior PP-based interventions to assess efficacy among midlife persons, (Feig et al., 2020) and developing infrastructure for text message delivery (Legler, Celano, Beale, Hoeppner, & Huffman, 2020). We chose to use MI given that it has been used to promote physical activity in a very wide range of settings and populations, but added specific content around stress reduction (in Sessions 5 and 6) along with time management and managing of potential caregiving, employment, or financial barriers (in Session 4 and throughout, with specialized content provided to participants as needed via an Appendix in the interventionist manual) (Huffman et al., 2021b; Kopf et al., 2014; Park et al., 2013a). This work was aimed to ensure that the MI-focused program had elements customized to the midlife population. This also included a prior proof-of-concept trial of a phone-only PP-MI intervention for midlife persons that found the program to be feasible and well-accepted (Huffman et al., 2021b). We next chose to conduct a second proof-of-concept trial of this PP-MI intervention– now utilizing a shorter duration of full phone sessions and with the addition of adjunctive text messaging, in an attempt to further optimize scalability. The framework utilizing the specific PP activities, MI-based physical activity program, and text messages was adapted from our prior intervention studies using a similar framework, (Huffman et al., 2020; Huffman, Feig, et al., 2019) other PP-based studies in medical populations, (Cohn et al., 2014; Moskowitz et al., 2012; Moskowitz et al., 2017) and the above developmental work to allow specific adaptation to the midlife population. Within these models, the main focus of the study was to assess feasibility of recruitment, retention, study procedures, and the phone and text components of the intervention (Gomez-Bernal et al., 2019).

**Inclusion and exclusion criteria.** To be included, participants were required to be *midlife adults* (age 45–64 at enrollment) and to have *low self-reported physical activity* (<150 min/week of moderate to vigorous physical activity [MVPA] over the prior week, as assessed via the short-form version of the validated International Physical Activity Questionnaire [IPAQ] scale Booth et al., 2003). This version of the scale has been associated with an overestimation of physical activity in some cases; (Lee, Macfarlane, Lam, & Stewart, 2011) given this, we felt that if participants self-reported low physical activity on this screening tool, it was rather likely that their true activity was quite low. Furthermore, we utilized the IPAQ-SF, because both the long-form and shorter form of the IPAQ have been validated and used in a wide variety of populations, (Craig et al., 2003; Sember et al., 2020) including patients at elevated cardiac risk, (Pfaeffli, Maddison, Jiang, Dalleck, & Lof, 2013) because it is very brief and simple to administer (and for participants to understand), and because it allows collection of physical activity data regarding a wide range of activities, including those that may not be picked up by accelerometer.

Exclusion criteria included: (a) a *condition preventing MVPA completion* (e.g. chronic obstructive pulmonary disease, osteoarthritis); (b) no access to a telephone or text messaging; (c) inability to communicate in English; (d) cognitive impairment, as assessed via a cognitive screen designed to assess appropriateness for research participation; (Callahan, Unverzagt, Hui, Perkins, & Hendrie, 2002) (e) *ongoing participation in physical activity or psychological programs* (e.g. mind-body interventions); and (f) coronary artery disease (CAD), defined as a prior acute coronary syndrome (myocardial infarction or unstable angina) or coronary stenosis identified through cardiac catheterization (i.e. 50% stenosis of the left main artery or 70% stenosis of another coronary artery). The age, communication, and medical condition (e.g. CAD) criteria were assessed via both the electronic record review and phone screen assessments; the remainder were assessed via phone screens. We excluded persons with CAD because one eventual goal of the program is to prevent heart disease and because individuals with CAD may have greater restrictions on moderate or vigorous activity.

Potential participants were identified via IRB-approved searches of our medical center’s electronic medical record system for midlife persons receiving care in affiliated primary care practices. Patients who had agreed to receive communications about ongoing research studies were sent opt-out letters from the study team, and those who did not opt out of contact completed a screening phone call. At the screening call, study staff described the project to potential participants and screened them for study criteria (e.g. low physical activity). Interested and eligible patients were mailed a study information fact sheet and were scheduled for a baseline phone study session, during which they provided informed consent and completed baseline self-report instruments. Participants were then mailed an accelerometer for physical activity assessment, wore it for one week, and then mailed it back to study staff.

After confirming adequate accelerometer wear time (at least 4 days with 10 + hours of wear time), a second study phone session was scheduled, and participants were mailed a written treatment manual and pedometer. During this session, participants spoke with a study interventionist, who introduced them to the treatment manual and pedometer, reviewed the program’s structure and rationale, and conducted an introductory session. If the participant had inadequate wear time, the study team would have mailed the accelerometer back to the participant for further wear, and when it was returned by mail a second time (and once adequate wear time was confirmed), the second phone session would have been completed (this was not required in this study, as all participants had adequate time on the first wearing).

The balance of the intervention was delivered by phone and text messages, with weekly phone contacts for 12 weeks (6 weeks of 30-minute phone sessions followed by 6 weeks of briefer [5-minute] phone check-ins) and ongoing weekly text messaging over the 12 weeks. Following the intervention, participants completed a follow-up assessment and received accelerometers by mail, wore them for 1 week, then returned them by mail.

I**ntervention*.*** Over the first 6 weeks of the program (7 sessions over 6 weeks, including the introductory session)**,** participants completed assigned intervention activities (e.g. PP exercises and physical activity goals) on their own, then participated in 30-minute weekly phone sessions with a study psychologist interventionist. The intervention consisted of a PP component that focused on the development of skills to promote psychological well-being in daily life, along with an MI component that utilized MI concepts and specific goal-setting strategies to increase engagement in physical activity. During the calls, the participant and interventionist reviewed the prior week’s activities, discussed the use of well-being skills, reviewed facilitators and barriers to physical activity, and discussed the assigned activities and rationale for the upcoming week (see Tables 1 and 2 for additional intervention detail, along with Appendix for full treatment manual).

**Table 1.** Initial PP-MI phone intervention content (7 phone sessions over 6 weeks)

Session	PP component	MI component
1 (in person)	*Gratitude for positive events* (Emmons & McCullough, 2003; Seligman et al., 2005) Participants identify and reflect on three positive events that occurred in the past week.	*Moving for better health/activity tracking* Participants report their current activity level, set an overall activity goal, discuss the importance and confidence in making the change, and consider the pros/cons of changing their activity.
2	*Gratitude letter* (Seligman et al., 2005) Participants write a letter of gratitude thanking a person for their kindness.	*Setting a SMART physical activity goal* Participants learn about and set a SMART (specific, measurable, attainable, relevant, and time-based) activity goal.
3	*Recalling past success* (Selimbegovic, Regner, Sanitioso, & Huguet, 2011) Participants recall a successful past life event and then write about the event, their personal contribution to the success, and positive feelings elicited by recalling it.	*Barriers and problem solving* Participants consider barriers and facilitators to being more physically active.
4	*Using personal strengths* (Seligman et al., 2005) Participants find a specific new way to use one of their ‘signature strengths’ in the next 7 days.	*Resources for activity:* Participants identify neighborhood, social, and equipment resources that can help them achieve physical activity goals.
5	*Enjoyable and meaningful activities* (Peterson, Park, & Seligman, 2005) Participants complete an enjoyable activity alone, an enjoyable activity with another person, and an activity that is more deeply meaningful to them.	*Stress reduction session 1* Participants learn and utilize new techniques focused on problem-oriented coping skills.
6	*The ‘Good Life’* (Seligman et al., 2005) Participants write about what their ‘good life’ would look like in the future in one or more life domains.	*Stress reduction session 2* Participants learn and utilize new techniques focused on emotion-focused coping skills.
7	*Skills application + future planning* Participants make a plan for continuing to use their PP-based skills in the future.	*Reviewing progress/considering the future* Trainers assist participants with reviewing their accomplishments and help them to create a plan for physical activity for the near future.

Each week, as part of the MI-informed behavior change portion of the program, prior to reviewing the specific weekly topics noted above, interventionists reviewed progress on the prior week’s activity goal, discussed intra-activity positive affect, used the 5A’s approach, tracked activity, and problem-solved cognitive or instrumental barriers to activity.

**Table 2.** One-way text messages (weeks 1-6).

Session	Session Topic	Component	Message
1	Gratitude for Positive Events	PP	One way to increase gratitude is to deliberately take note of small positive things that happen. This week, think about and write down three good things that happened!
1	Moving for Better Health	MI	Keeping track of your physical activity can be very helpful to maintain motivation. Try using a notebook, spreadsheet, smartphone app, or whatever works best for you!
2	Expressing Gratitude	PP	Almost half of your happiness is under your control. This week, write a thank you letter for something someone did for you. It can lift your spirits (and theirs)!
2	SMART goals	MI	Setting reachable goals can be really helpful in establishing a new routine. Try setting a new exercise goal today that is do-able based on your fitness level.
3	Remembering past success	PP	Remembering your past successes is not always easy but can increase happiness. This week, remind yourself of your strengths by visualizing a past success!
3	Barriers	MI	Barriers, such as a lack of time, can make it hard to reach your goals. If you struggle to find enough time to be active, try spreading your activity throughout the day.
4	Using strengths	PP	This week, focus on one of your strengths and use it in a new way. Pick an activity you might not usually do and use your strength to make it happen. It can feel great!
4	Resources	MI	Resources like family, parks, walking trails, and even comfortable clothes and shoes can help you be active. This week, use your resources to reach your activity goals!
5	Enjoyable and meaningful activities	PP	A great way to boost your mood is to engage in fun or meaningful activities. This week, set aside 15 min for yourself to do something enjoyable or meaningful!
5	Stress reduction (problem-focused coping)	MI	Sources of stress can get in the way of being active. This week, use problem-solving strategies to reduce stress and meet your physical activity goal!
6	The Good Life	PP	This week, spend some time thinking about what a ‘good life’ would look like for you. Then, think about what you can do this week to start to make this life a reality!
6	Stress reduction (emotion-focused coping)	MI	Emotion-focused coping strategies, like deep breathing or visualization, can help reduce stress and put you in the right frame of mind for exercise. Try them this week!
7	Conclusion	Conclusion	Thank you again for participating in MASTERY. Your trainer will continue to communicate using text messages to help you meet your goals moving forward!

*Phone sessions in the first 6 weeks.* The PP program contained specific activities (see Table 1 and next paragraph below), selected via review of effective PP activities in prior research (Seligman et al., 2005; Sin & Lyubomirsky, 2009) and our qualitative work focused on well-being in midlife persons (Madva et al., 2018). The framework for sessions—reviewing prior weeks’ activities from the treatment manual, considering skill application, and introducing the subsequent activity—was based on the frameworks used in our prior projects and other PP-based interventions in medical settings (Moskowitz et al., 2012; Moskowitz et al., 2017). This component was specifically customized for midlife adults, based on our review of midlife physical activity literature (Spiteri et al., 2019; Zou et al., 2021) and a prior midlife study conducted by our team, (Huffman et al., 2021b) by providing potentially relevant examples and context (e.g. juggling numerous responsibilities, caring for family) and selecting activities that seemed most specific to midlife (e.g. the activity on meaningful activities was selected because many midlife persons begin to focus on meaning, purpose, and legacy more distinctly during this life phase Lachman, 2004). Participants completed specific activities outlined in the treatment manual each week and wrote about the activity and its effects in the manual.

**Figure 2.**
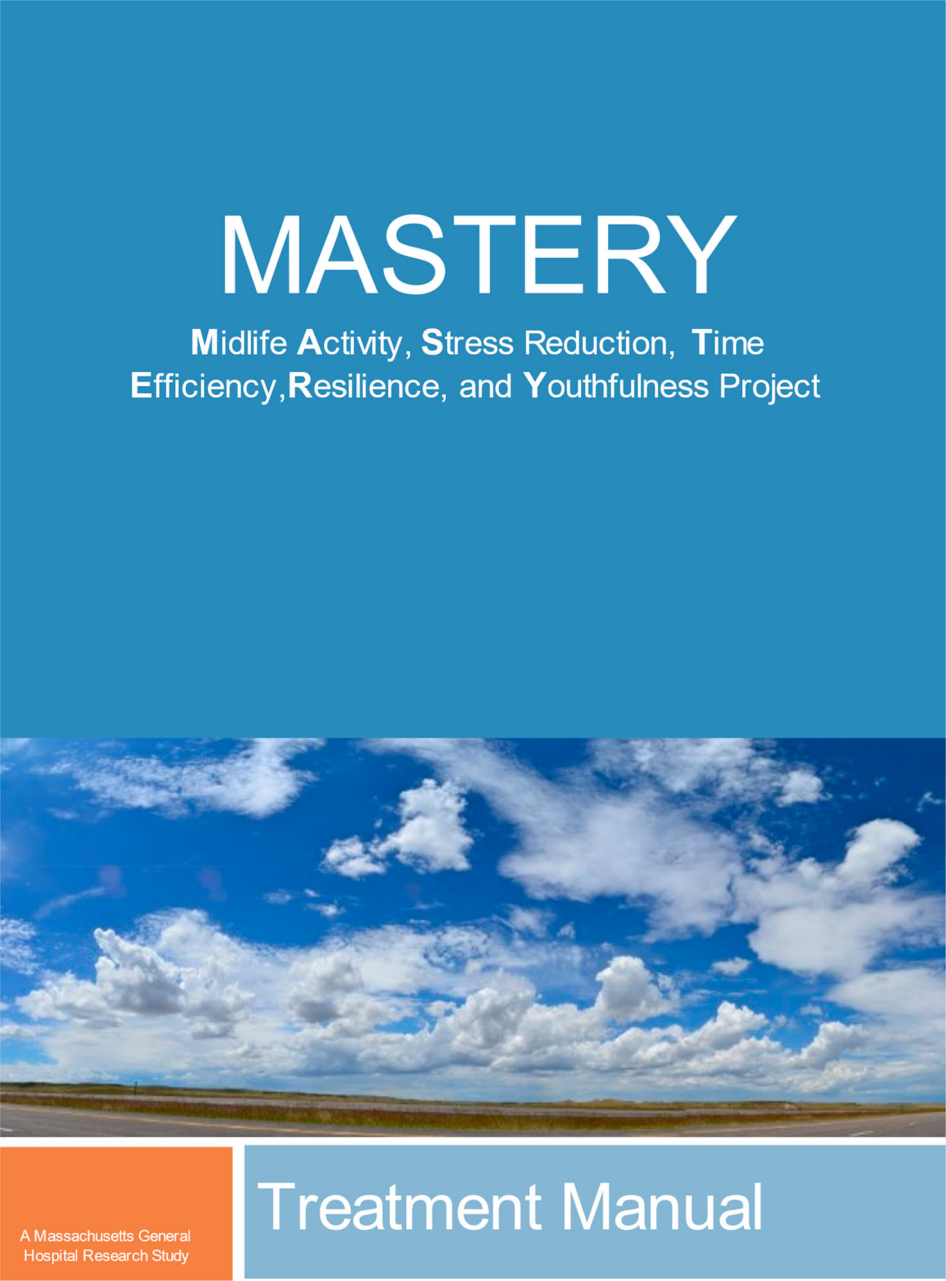
Pages from the PP-MI treatment manual.

During the PP portion (∼10 min) of phone sessions during the first 6 weeks of the program, participants and interventionists reviewed the prior week’s activity, discussed how skills from that activity could be utilized in daily life, and discussed the rationale for the next week’s PP activity. The PP exercises were as follows*: gratitude for positive events* (Session 1), *expressing gratitude* (Session 2), *recalling past success* (Session 3), *using personal strengths* (Session 4), *enjoyable and meaningful activities* (Session 5), *imagining the ‘good life’* (Session 6) and, *planning for the future* (Session 7; focused on translating skills to daily life). Further details for each activity and sample pages from the manual are in Table 1 and Figure 2, respectively, with the full treatment manual included as an Appendix.

The midlife-adapted MI portion (∼15 mins) of phone sessions in the first 6 weeks assessed motivation to engage in physical activity, discussed barriers to activity, and set physical activity goals or used MI tools to boost motivation, depending on the participant’s stage of change. This component also included a brief stress reduction module based on feedback in our prior work in midlife adults (Huffman et al., 2021b; Madva et al., 2018). Each week, the ‘5A’s’ (Ask, Advise, Assess, Assist, Arrange) practice framework was utilized, and at each session interventionists and participants reviewed the prior week’s physical activity goal, assessed stage of change, and problem-solved barriers to being active. Each week, a distinct topic (e.g. tracking physical activity, using resources to facilitate activity) was also discussed with participants (see Table 1). The phone session ended with the setting of a concrete physical activity goal for the following week.

The initial four sessions of the MI component introduced specific MI-based concepts and skills for boosting physical activity, including a focus on participants’ assignment of importance and confidence in changing their activity level, means of tracking activity, and utilizing the ‘SMART’ (specific, measurable, attainable, relevant, and time-based) framework (Doran, 1981) for setting and reaching goals. The next two sessions utilized a stress reduction module customized to midlife, adapted from established stress reduction programs and our prior work with midlife persons (Huffman et al., 2021b; Kopf et al., 2014; Park et al., 2013a). The final session (Session 7), in parallel with the PP component, reviewed program content and progress and helped participants to set specific, realistic goals for physical activity.

*Phone check-ins in the final 6 weeks.* The phone check-ins (∼5 min) in the final 6 weeks of the program were structured calls with participants that focused on goals for PP skill use and physical activity completion that were created at the final phone session (Session 7) and via weekly text messaging (see below). The calls, performed between the goal-focused text messaging that occurred each week in the final 6 weeks of the program, reviewed participant PP and activity goals, checked in about progress, reviewed facilitators and barriers to completion, and modified goals if needed. Interventionists also inquired about specific sources of stress common in midlife (e.g. occupational, caregiving, and financial stress) and referred participants to resources and strategies in the Appendix of their treatment manual, when relevant.

*Text messaging.* Text messaging was utilized to supplement the phone sessions and written treatment manual content. In the first 6 weeks of the program, one-way messages were sent to participants that reinforced content from the phone sessions and treatment manual. For example, following Session 1 (which focused on recalling positive life events in the PP component and tracking physical activity in the MI component), the text messages sent to participants were:

(PP message) *One way to increase gratitude is to deliberately take note of small positive things that happen. This week, think about and write down three good things that happened!*

(MI message) *Barriers, such as a lack of time, can make it hard to reach your goals. If you struggle to find enough time to be active, try spreading your activity throughout the day.*

The messages were sent utilizing an IRB-approved, HIPAA-compliant approach utilizing messages selected from our Amazon Web Services DynamoDB message database (Amazon Web Services, 2020) and deployed via the Twilio (Twilio, 2021) program that sends messages via a centralized phone number, rather than an individual cell phone. We have utilized these platforms and this approach in prior text message intervention studies (Legler et al., 2018; Legler et al., 2020). See Table 2 for further examples of text messages.

In the final six weeks of the program, participants completed brief two-way text message sessions with study interventionists. These sessions, performed between the weekly goal-focused phone check-ins, allowed participants to review progress towards their PP and activity goals for the week, discuss any challenges completing the goals with the interventionist, and modify their goals if needed. These messages were sent by the participant’s assigned study interventionist from a study-specific phone number via Twilio in a HIPAA-compliant, IRB-approved manner.

*Behavior change techniques.* Overall, using the framework of Michie and colleagues, (Michie et al., 2013) this physical activity program utilized the following behavior change techniques: self-monitoring of behavior, review of outcome goal, discrepancy with current behavior, problem-solving/coping planning, review of behavior goals, and goal-setting, making this intervention more than a simple MI intervention but instead an MI-informed physical activity behavioral intervention. Furthermore, the text message component of the intervention, given the nature of this modality, was largely focused on providing direct education, facilitating goal setting, and providing more direct advice than would be utilized in a traditional MI intervention, making this component a broader behavioral change intervention.

*Intervention delivery and fidelity.* Interventionists were four psychologists (three of whom were board-certified, while the other was a research psychologist) who received training in both the PP and MI components of the intervention from the lead study intervention supervisor (CC), based on a protocol from our prior work (Huffman et al., 2020; Huffman, Feig, et al., 2019; Zambrano et al., 2020a). Training consisted of didactic sessions, provision of written materials (including a written annotated training manual containing guidelines and tips for each session), and role-playing sessions, which were based on similar training programs run by our behavioral intervention collaborators (Park et al., 2006; Park et al., 2013b). Throughout the trial, interventionists attended weekly hour-long intervention supervisory meetings with this supervisor experienced in the delivery of PP and MI interventions to review cases and assess fidelity to the study intervention. Sessions were audiotaped, and supervision sessions included a review of portions of the audiotaped sessions. A study-created fidelity scale was utilized to assess fidelity in the cases reviewed at the session, and feedback was provided to the interventionists. We aimed to conform to best practices in behavioral intervention treatment fidelity as outlined by the NIH Change Consortium checklist (Borrelli et al., 2005) related to study design, provider training, treatment delivery, treatment receipt, and enactment of treatment skills; we did not formally record number of minutes per session (though reviewed time during supervision meetings), and did not formally develop an assessment of participants’ ability to perform the related skills, though this was a major focus of the supervisory meetings, and we adhered to the other recommendations in the checklist.

Overall, if implemented, the program would require a clinician to deliver the intervention (we have utilized bachelor’s level-clinicians, social workers, nurses, and psychologists for PP-based programs in prior work), a written treatment manual, the infrastructure for sending text messages, and adequate time for conducting the weekly initial sessions (∼25 min) in the first 6 weeks and briefer phone and text check-ins (∼5 min each) in the next 6 weeks, along with additional time to account for missed calls and documentation.

**Study assessments and outcomes.** Participants’ baseline sociodemographic and medical characteristics were obtained via patient report and medical record review. For measures of feasibility and acceptability, session completion and participant ratings of sessions were recorded by study interventionists. Finally, participants completed self-report measures at baseline and 12 weeks via telephone and wore accelerometers at both time points for objective physical activity outcomes.

Primary study outcomes: feasibility and acceptability. We measured feasibility via *rates of phone session completion* (both full phone session and phone check-in; full completion required attending the phone call, completion of the prior week’s planned PP activity/skill, and setting a new physical activity goal). The two primary feasibility measures were: (a) the proportion of all possible sessions that were completed across all participants, and (b) the number of participants completing a majority of phone sessions. We chose these to assess the overall scope of session completion and to have a clinically relevant metric, as we deemed that completing a majority of phone sessions would convey the key elements of intervention content to the participant; we have used these metrics in prior feasibility studies of behavioral interventions (Celano et al., 2019; Huffman et al., 2015b). We measured acceptability via *participant ratings of phone session ease and utility* (0-10 Likert scale, 0 = not easy/useful, 10 = very easy/useful) of both the PP and MI activities/content each week during intervention calls over the initial 7 phone sessions, for a total of 4 weekly ratings.

We also collected *secondary* measures of acceptability via self-report assessments at 12 weeks regarding the utility of each study component (phone sessions, phone check-ins, one-way text messages, and two-way text sessions; 0–10 Likert scales).

Secondary study outcomes: Accelerometer-measured physical activity and self-report measures. This initial proof-of-concept study was not designed (and is not appropriate) to assess intervention efficacy given the small sample and lack of control condition, and for this reason the outcomes noted below are exploratory. We assessed physical activity (mean *minutes of MVPA* and *total steps* per day, with MVPA serving as the main secondary outcome measure) via the well-validated Actigraph GT3X+ accelerometer (Aadland & Ylvisaker, 2015). MVPA, measured by accelerometer, was chosen as the main secondary outcome because it is the intensity of activity most clearly and strongly associated with lower cardiovascular risk and overall improved health outcomes (Colberg et al., 2016; Full et al., 2021; Kraus et al., 2019; Stamatakis et al., 2019). We required valid accelerometer data from at least 10 h per day for at least 4 days as per prior studies using these devices and a study of the device compared to doubly labeled water (Helgadottir, Forsell, & Ekblom, 2015; Huffman et al., 2020). We did not include one weekend day of activity, given that we expected that not all participants (or midlife persons) would working, or that some would not be working traditional daytime weekday positions. For MVPA, we utilized a threshold of 1952 counts/minute as per prior work (Cain, Conway, Adams, Husak, & Sallis, 2013).

For self-report outcomes, we assessed *positive affect* using the positive affect items from the Positive and Negative Affect Schedule (PANAS; Crawford & Henry, 2004) *optimism* using the Life Orientation Test-Revised (LOT-R) scale, (Scheier, Carver, & Bridges, 1994) and *anxiety and depression* via the Hospital Anxiety and Depression Scale (HADS) (Bjelland, Dahl, Haug, & Neckelmann, 2002). As potential mediators of the effect of PP content on physical activity, we assessed *internal locus of control* via the Multidimensional Health Locus of Control (MHLC) scale, (Wallston, 2005) perceived social support via the Multidimensional Scale of Perceived Social Support (MSPSS; Zimet, Powell, Farley, Werkman, & Berkoff, 1990) and *physical activity self-efficacy* via the Self-Efficacy for Exercise (SEE) measure (van der Heijden, Pouwer, & Pop, 2013). We assessed *physical function* using the PROMIS 20-item physical function (PF-20) scale, (Jensen et al., 2015) and we measured *health-related quality of life* (HRQoL) via the Medical Outcomes Study Short Form-12 (SF-12) scale (Ware, Kosinski, & Keller, 1996). Finally, we also assessed *self-reported health behaviors*, including physical activity via the International Physical Activity Questionnaire (IPAQ) (Lee et al., 2011) and overall *adherence to healthy diet, physical activity, and medications* using items from the Medical Outcomes Study Specific Adherence Scale (MOS SAS) (DiMatteo, Hays, & Sherbourne, 1992).

**Statistical analysis.** Descriptive statistics (proportions and means/SD) were used to report session completion rates and ease/utility scores. As a threshold for feasibility, based on our prior work. (Huffman, Feig, et al., 2019; Huffman et al., 2020) we utilized *a priori* metrics of 70% completion of phone sessions/check-ins across all participants and more than half of participants completing a majority of the 12 phone sessions. For our primary measures of acceptability regarding the phone session ease and utility, we calculated mean ease and utility ratings of each session component (i.e. PP and MI) across all participants and utilized mean scores of greater than 7.0 as our *a priori* threshold for acceptability, based on prior work using similar ratings for acceptability of behavioral interventions (Huffman, Feig, et al., 2019; Huffman et al., 2020). We also utilized this cutoff for our secondary measures of acceptability measured at 12 weeks with more global assessments of the utility of the phone sessions, phone check-ins, one-way texts, and two-way texts.

For pre-post changes in physical activity and self-report outcomes, we used mixed effects regression models, with a categorical effect of time ; these models allow inclusion of all participants, even those with missing data, in an intent-to-treat model. No covariates were used in the model for this initial feasibility study. For these pre-post analyses, we focused primarily on the effect size (ES) of the change in the measure, calculated as the coefficient from the mixed model divided by the SD of the residual for the measure, rather than statistical significance, given the secondary nature of these outcomes in this small sample. In a further set of analyses, we examined correlations between number of sessions completed and change in the secondary outcomes (e.g. MVPA, positive affect). All analyses were conducted using Stata version 15.1 (StataCorp; College Station, TX).

## Results

**Figure 3.**
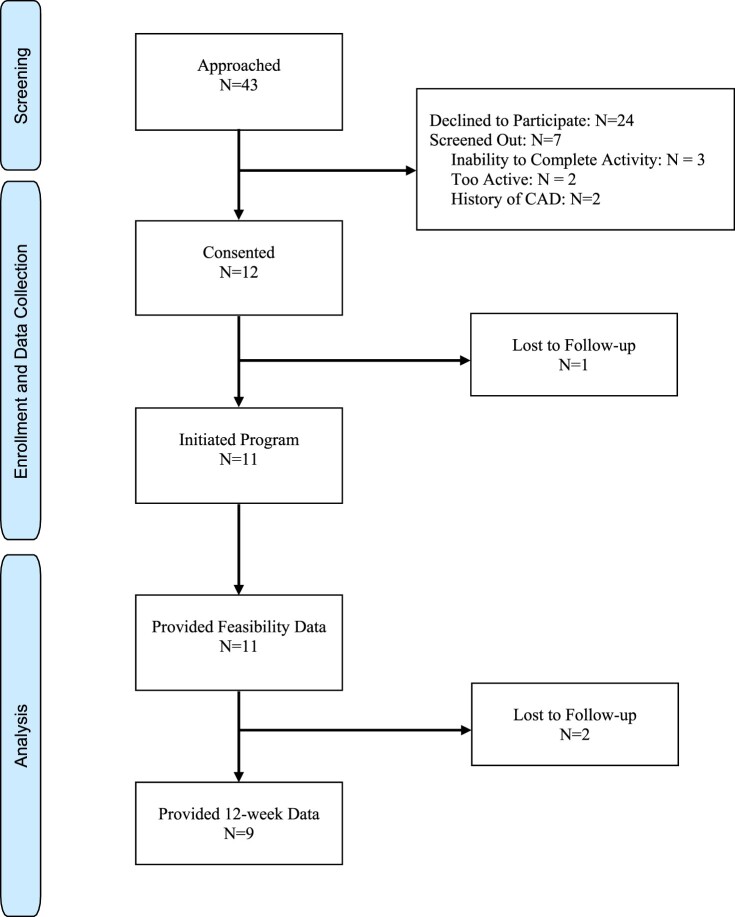
CONSORT Study Flow Diagram

We contacted 43 patients, and after hearing about the study and undergoing relevant screening, 19 were interested in participating. Seven screened out, and a total of 12 participants enrolled in the proof-of concept trial (see study flow diagram, Figure 3 for more detail). Of these 12 participants, 11 provided feasibility data, and 9 provided all follow-up data. The mean age of participants was 56.1 (SD 5.8) years, 9 (75%) were women, and 8 (67%) were non-Latino White persons; Table 3 displays all participant baseline sociodemographic and medical characteristics. Participants at baseline had a mean of 105 min of MVPA/week as measured by accelerometer. Those who did not follow up (n = 3; all women, mean age 56.0) were very similar in baseline characteristics to those who remained in the study. They also had slightly lower (mean MVPA/week = 100 min) baseline MVPA compared to the overall sample (105 min). Fidelity was high across all interventionists as rated at supervision meetings.

**Table 3.** Participant characteristics.

Characteristic	Overall
*Sociodemographic characteristics (N (%) unless otherwise noted)*	
Age (M [SD])	56.2 (5.8)
Female sex	9 (75)
Non-Hispanic White	8 (66.7)
*Medical characteristics/cardiac risk factors (N (%))*	
Diabetes	2 (16.7)
Hyperlipidemia	6 (50)
Hypertension	7 (58.3)
Current smoking	1 (8.3)
*Baseline self-report and physical activity measures (M (SD))*	
Positive affect (PANAS) (range 10-50)	30.3 (5.2)
Dispositional optimism (LOT-R) (range 0-24)	16.3 (4.5)
Depression (HADS-D) (range 0-21)	6.1 (2.8)
Anxiety (HADS-A) (range 0-21)	8.8 (3.6)
Self-efficacy for exercise (SEE) (range 0-90)	56.1 (18.0)
Internal locus of control (MHLC) (range 6-36)	24.8 (4.4)
Mental HRQoL (SF-12 MCS) (range 0-100)	42.9 (10.9)
Physical HRQoL (SF-12 PCS) (range 0-100)	48.6 (9.7)
Physical function (PROMIS PF-20) (range 0-100)	93.5 (7.9)
Adherence (MOS SAS) (range 3-18)	10.7 (2.6)
MVPA (minutes/day; GT3X+)	15.0 (9.2)
Total daily steps (GT3X+)	5455.1 (2880.4)
Self-reported physical activity (IPAQ)	2581.8 (3834.7)

*p < .05

**Legend.** HADS = Hospital Anxiety and Depression Scale (HADS-A = Anxiety Subscale, HADS-D = Depression); HRQoL = health-related quality of life; IPAQ = International Physical Activity Questionnaire; LOT-R = Life Orientation Test, Revised; MHLC = Multidimensional Health Locus of Control scale; MOS SAS = Medical Outcomes Study Specific Adherence Scale; MSPSS = Multidimensional Scale of Perceived Social Support; MVPA = Moderate to vigorous physical activity; PANAS = Positive And Negative Affect Schedule; PF-20 = PROMIS Physical Function 20-item measure; SEE = Self-Efficacy for Exercise scale; SF-12 MCS = Medical Outcomes Study Short Form-12 Mental Component Score; SF-12 PCS = Medical Outcomes Study Short Form-12 Physical Component Score. Internal locus of control scale

Regarding feasibility, participants (including immediate dropouts) completed a mean of 9.4 (SD 4.8) sessions (113/144 possible total sessions [78.4%] completed), with 9 (75%) participants completing more than half of all possible sessions (i.e. 7 + out of 12). For acceptability, participants’ mean ratings of PP-MI phone session ease and utility ranged from 7.9-9.2 out of 10, with all components exceeding our a priori acceptability threshold (PP ease = 7.9 [SD 2.4], PP utility = 9.2 [SD 1.6], MI ease = 9.2 [SD 1.4], MI utility = 9.2 [SD 1.4]).

On additional measures of acceptability obtained at the 12-week follow-up, participants rated the overall utility of full phone sessions (first 6 weeks) 9.0/10 (SD 1.4), phone coaching check-ins (final 6 weeks) 8.4/10 (SD 2.8), one-way text messages (first 6 weeks) 6.6/10 (SD 3.7), and check-in texts (final 6 weeks): 9.3/10 (SD 1.2).

Regarding the secondary study pre-post outcomes (Tables 4 and 5), participants reported increases in both self-reported and accelerometer-measured physical activity over time. On the main secondary outcome of accelerometer-measured MVPA, participants demonstrated medium magnitude ES improvements (mean increase of 7.2 min [residual SD 11.6] per day; *p* = .19; ES = .62). Participants also had a small ES magnitude improvement on accelerometer-measured steps per day (mean increase of 431 [SD 1886] steps per day); *p* = .64; ES = .23. On self-reported physical activity measured via the IPAQ, participants had a large ES improvement in activity (mean 2611 MET-minute improvement [SD 1130], *p* < .001; ES = 2.31).

**Table 4.** Secondary outcomes: pre-post changes in study outcome measures.

*Pre-post changes in self-report and physical activity outcomes (Secondary Aim)*					
Measure	12 weeks				
	EMD	SE	z	*P*	ES
*Accelerometer-measured physical activity outcomes*					
MVPA (mean min/day)	7.20	5.51	1.31	.19	.62
Mean total daily steps	431.1	913.9	0.47	.64	.23
*Psychological outcomes*					
Positive affect (PANAS)	5.41	2.56	2.11	.035*	.94
Dispositional optimism (LOT-R)	2.38	1.84	1.29	.20	.57
Depression (HADS-D)	−3.64	1.07	−3.38	.001*	1.49
Anxiety (HADS-A)	−2.84	1.08	−2.62	.009*	1.20
Internal locus of control (MHLC)	3.74	0.90	4.16	<.001*	1.93
Perceived social support (MSPSS)	-.49	2.83	−0.17	.86	.08
Exercise self-efficacy (SEE)	8.88	5.19	1.71	.087	.79
*Additional functional/behavioral measures*					
Mental HRQoL (SF-12 MCS)	6.55	3.77	1.74	.082	.78
Physical HRQoL (SF-12 PCS)	0.25	2.04	0.12	.90	.06
Physical function (PROMIS PF-20)	3.81	1.55	2.46	.014*	1.14
Self-reported physical activity (IPAQ)	2610.7	530.3	4.92	<.001*	2.31
Self-reported adherence (MOS SAS)	4.16	0.73	5.73	<.001*	2.81

* *p* < .05

**Legend.** ES = effect size; HADS = Hospital Anxiety and Depression Scale (HADS-A = Anxiety Subscale, HADS-D = Depression); HRQoL = health-related quality of life; IPAQ = International Physical Activity Questionnaire; LOT-R = Life Orientation Test, Revised; MI = Motivational interviewing; MHLC = Multidimensional Health Locus of Control scale; MOS SAS = Medical Outcomes Study Specific Adherence Scale; MSPSS = Multidimensional Scale of Perceived Social Support; MVPA = Moderate to vigorous physical activity; PANAS = Positive And Negative Affect Schedule; PF-20 = PROMIS Physical Function 20-item measure; P*P* = Positive psychology; SF-12 MCS = Medical Outcomes Study Short Form-12 Mental Component Score; SF-12 PCS = Medical Outcomes Study Short Form-12 Physical Component Score.

**Table 5.** Secondary outcomes: pre-post changes in study outcome measures.

*Pre and Post Values for Outcome Measures in Secondary Efficacy Analyses*				
Measure	Baseline		12 weeks	
	Mean	SD	Mean	SD
*Accelerometer-measured physical activity outcomes*				
MVPA (mean min/day)	15.0	13.4	22.2	15.6
Mean total daily steps	5455.1	2823.1	5886.2	3169.0
*Psychological outcomes*				
Positive affect (PANAS)	30.3	6.1	35.7	7.1
Dispositional optimism (LOT-R)	16.3	4.4	18.6	5.0
Depression (HADS-D)	6.1	2.4	2.4	2.8
Anxiety (HADS-A)	8.8	3.6	5.9	4.0
Internal locus of control (MHLC)	24.8	4.1	28.6	4.4
Perceived social support (MSPSS)	67.1	14.3	66.6	15.0
Exercise self-efficacy (SEE)	56.1	18.6	65.0	20.3
*Additional functional/behavioral measures*				
Mental HRQoL (SF-12 MCS)	42.9	9.7	49.4	11.1
Physical HRQoL (SF-12 PCS)	48.6	10.1	48.9	10.6
Physical function (PROMIS PF-20)	93.5	6.6	97.3	7.1
Self-reported physical activity (IPAQ)	2581.8	4362.8	5192.5	4456.1
Self-reported adherence (MOS SAS)	10.7	2.9	14.8	3.2

**Legend.** ES = effect size; HADS = Hospital Anxiety and Depression Scale (HADS-A = Anxiety Subscale, HADS-D = Depression); HRQoL = health-related quality of life; IPAQ = International Physical Activity Questionnaire; LOT-R = Life Orientation Test, Revised; MHLC = Multidimensional Health Locus of Control scale; MOS SAS = Medical Outcomes Study Specific Adherence Scale; MSPSS = Multidimensional Scale of Perceived Social Support; MVPA = Moderate to vigorous physical activity; PANAS = Positive And Negative Affect Schedule; PF-20 = PROMIS Physical Function 20-item measure; SF-12 MCS = Medical Outcomes Study Short Form-12 Mental Component Score; SF-12 PCS = Medical Outcomes Study Short Form-12 Physical Component Score.

Participants also had medium to large ES magnitude improvements (ES = .57-1.49; Table 4) on psychological metrics, including positive affect, optimism, depression, and anxiety, with similar improvements in psychological factors that could mediate physical activity change (self-efficacy for exercise, internal locus of control; ES = .79-1.94), though no beneficial effect on perceived social support and somewhat more mixed effects on function and HRQoL (Table 4). There was a very large improvement in self-reported adherence to diet, activity, and medications via the MOS SAS (mean improvement 4.16 (SD 1.48); *p* < .001; ES = 2.81).

On examination of the relationship between number of sessions completed and secondary efficacy outcome measures (Table 6), we in general found that completing a greater number of sessions was associated with greater improvements in outcomes. For example, there were small-medium correlations between number of sessions completed and improvements in MVPA (r = .30) and number of steps taken (r = .46); the greatest correlations were between session completion and self-efficacy for exercise (r = .50) and overall self-reported adherence to health behaviors (r = .68), though none of these correlations were statistically significant. In contrast, there were negative correlations between number of sessions completed and some secondary outcomes, including perceived social support, locus of control, and physical health-related quality of life.

**Table 6.** Relationships between number of intervention sessions completed and improvements in secondary outcome measures.

Outcome measure	r	*p* value
MVPA	.30	.48
Steps/day	.43	.29
PANAS	.32	.41
LOT-R	.18	.64
HADS-D	-.19	.62
HADS-A	.11	.78
MHLC	-.44	.24
MSPSS	-.21	.58
SEE	.50	.17
SF-12 MCS	.41	.28
SF-12 PCS	-.11	.77
PROMIS PF-20	.33	.38
IPAQ	.37	.33
MOS SAS	.68	.06

**Legend.** HADS = Hospital Anxiety and Depression Scale (HADS-A = Anxiety Subscale, HADS-D = Depression); HRQoL = health-related quality of life; IPAQ = International Physical Activity Questionnaire; LOT-R = Life Orientation Test, Revised; MHLC = Multidimensional Health Locus of Control scale; MOS SAS = Medical Outcomes Study Specific Adherence Scale; MSPSS = Multidimensional Scale of Perceived Social Support; MVPA = Moderate to vigorous physical activity; PANAS = Positive And Negative Affect Schedule; PF-20 = PROMIS Physical Function 20-item measure; SEE = Self-Efficacy for Exercise Scale; SF-12 MCS = Medical Outcomes Study Short Form-12 Mental Component Score; SF-12 PCS = Medical Outcomes Study Short Form-12 Physical Component Score.

## Discussion

In this initial trial, we found that a phone- and text-delivered, midlife-specific intervention to promote physical activity was feasible, well-accepted, and generally associated with medium-to-large ES magnitude improvements in accelerometer-measured MVPA and other clinically relevant outcomes. Regarding our primary aims of feasibility and acceptability, approximately 78% of all possible phone sessions were fully completed, and three-quarters of participants completed 7 or more of the 12 phone sessions. Likewise, ratings of the ease and utility of both the PP and MI components of the phone sessions had mean scores ranging from 7.9-9.2/10; these outcomes surpassed our *a priori* thresholds in all cases. On additional measures of acceptability obtained at the 12-week follow-up, participants rated highly the overall utility of full phone sessions, phone coaching check-ins, and check-in texts, but gave lower ratings for the one-way text messages; this relatively low rating for one-way text messages did not meet our *a priori* threshold for acceptability of 7.0/10.

Such results regarding session completion and intervention acceptability are consistent with previous studies of phone-based PP-MI interventions among midlife persons and those with medical illness (Huffman et al., 2021a; Huffman, Feig, et al., 2019; Huffman et al., 2020). This trial extends prior work, as it represents only the second study to our knowledge that has used this PP-based approach specifically in midlife persons (Huffman et al., 2021b). The prior study was a single-arm study in 11 participants examining a PP and MI-based approach to physical activity. The experimental intervention utilized similar content and outcome measures, but was more intensive, as it utilized 12 weeks of phone sessions from psychologists. That study had somewhat larger effects on MVPA (*d *= .87 vs. *d *= .62 in this project) and similar but slightly higher scores on measures of feasibility and acceptability. This project extends that prior work, as it includes half the number of full phone sessions and is the first to utilize text messaging as an accessible means to provide additional support and content beyond the 6-week phone session period. Such work directly addresses the public health problem of low physical activity among the growing population of midlife persons, who are at risk for developing numerous chronic medical conditions during this life stage. This remote intervention approach might be particularly well-suited to this population, as in-person interventions are likely suboptimal given that many midlife adults must manage multiple competing demands and experience substantial time pressure, and requiring fewer full (∼25 min) phone sessions may be beneficial in terms of patient and provider burden. A prior, non-PP based, intervention for persons in midlife was effective in the short-term but not long-term, and it used in-person individual or group sessions, which may be less feasible (Ribeiro, Martins, & Carvalho, 2014). The intervention studied in this trial may thus have some distinct benefits from the above-noted prior work, though substantially more research on the program must be conducted before any such conclusions could be made.

Along with meeting benchmarks for feasibility and acceptability, the intervention was associated with improvements in physical activity, including MVPA, among this cohort of midlife adults with low physical activity. Physical activity, especially MVPA, is strongly linked with lower rates of cardiac disease, other chronic medical conditions, and mortality (Saint-Maurice et al., 2019). Likewise, participants showed substantial improvements in psychological factors such as positive affect and depression during the intervention. Improvements in these domains have the potential to improve quality of life, promote greater engagement in physical activity, and boost overall health, given the literature prospectively connecting improvements in these factors with greater participation in physical activity and superior health outcomes (Levine et al., 2021; Zambrano et al., 2020b). Indeed, in this study, participants moved from being a mean of 45 min below recommended weekly MVPA completion of 150 min per week (105 min/week at baseline on accelerometer) to exceeding this benchmark (mean of approximately 155 min/week, based on improvement by 7.2 min per day) by study end.

Several findings were notable. Though three-quarters of participants completed a majority of sessions, a small number dropped out shortly after enrollment, suggesting that simpler or shorter procedures, especially at the outset of the study, might improve initial engagement. While participants rated most intervention components highly, on secondary acceptability analyses, participants rated the utility of the one-way text messages in the first 6 weeks as somewhat lower than the subsequent two-way messages, suggesting that some form of live or automated interactivity may be an important aspect of text messaging in such an intervention. It may also be the case that the two-way messages were well-received in the context of an existing connection with the interventionist via the phone sessions and that such messaging, without such prior contact/familiarity, would be less well-accepted. Indeed, the literature on text message interventions does suggest that brief interactive phone or text-based contact or coaching with an interventionist as part of such interventions may substantially boost impact (Godino et al., 2016; Godino et al., 2019; Smith et al., 2020). In terms of mediating variables, internal constructs such as locus of control and self-efficacy, but not externally-focused perception of social support, improved during the intervention, suggesting that these may be factors that helped participants to become more physically active. Of these variables, self-efficacy may be particularly important, given the literature suggesting that self-efficacy for exercise is a major contributor to engaging in (and increasing) physical activity, and future studies should examine the mediating effects of this variable (Bauman et al., 2012; Locke, McMahon, & Brawley, 2020).

Scalability is a key issue for an intervention such as this one that is targeted at a major public health problem in a broad population of busy adults with multiple stressors. While we were not able to obtain information on those who declined participation, those participants who dropped out were largely similar to those who remained in the study. Furthermore, such an intervention may be more challenging for those with busy daytime work schedules plus family commitments (e.g. working parents) in terms of completion of phone sessions, while text messaging may be less familiar to some who use this modality less, including some adults on the older spectrum of midlife. Other factors that could contribute to participation and engagement in the program—either as a research project or in clinical implementation—could include current levels of motivation to change behavior, self-efficacy to change physical activity, and financial and time-related factors, all of which need to be assessed in future projects.

Finally, while there were small to medium improvements in accelerometer-measured physical activity, there were much larger improvements in self-reported activity and overall adherence. This could suggest that the accelerometers may not have picked up all forms of activity completed by participants (e.g. swimming), or instead that participants may have overestimated their improvements in these self-care domains. Indeed, there is substantial research examining differences in self-reported physical activity and objectively measured activity, with self-reported physical activity appearing to be overestimated in most cases (Ainsworth, Cahalin, Buman, & Ross, 2015). (Sallis & Saelens, 2000) The self-report measure used in this study, the short form of the IPAQ, has been specifically associated with overestimation of the amount of physical activity reported compared with objective measures (Lee et al., 2011; Rzewnicki, Vanden Auweele, & De Bourdeaudhuij, 2003). While this measure has been found to be reliable within participants, (Lee et al., 2011) and baseline activity as well as follow-up activity may have been overreported, this prior work suggests that the accelerometer-measured physical activity change seems much more likely to represent an accurate estimation of the effects of the intervention on this outcome.

This single-arm proof-of-concept trial had numerous limitations. For example, it utilized a small sample derived from a single urban academic medical center, study outcomes were assessed over only 12 weeks, and there was no control condition. Participant self-report instruments were administered by phone, which can lead to participants responses’ being affected by socially desirable responding or experimental demand. Likewise, the self-report physical activity measure used, as noted, has been associated with overestimation of physical activity, though this would presumably be true at both baseline and follow-up assessments. The effect size estimates obtained for changes in MVPA and other outcomes are highly speculative given the very small (and uncontrolled) sample in this proof of concept trial and should not be used to power future studies of this intervention. Longer, larger, well-controlled studies of the intervention are required to identify the clinical effectiveness of the program in front-line clinical settings.

In conclusion, in this proof-of-concept trial of a phone and text message PP-MI program adapted for inactive midlife adults, we found that the intervention was feasible, acceptable, and associated with improvements in MVPA and other clinically relevant measures. Further studies of the intervention are required to more definitively test the impact of this program, conduct rigorous analyses of mechanism and mediation of intervention effects to better understand how the program can work, and utilize implementation analyses that would be needed to estimate the scalability of the program.

## Declaration of interest and funding/support

Time for analysis and article preparation was also funded by the National Heart, Lung, and Blood Institute through grant (R01HL155301) (CMC), and by the National Institute for Nursing Research through grant R21NR018738 (JCH). The project was also supported by the National Center for Advancing Translational Science through grant 1UL1TR002541-01. The authors have no conflicts of interest to report related to this work. This study was presented in oral form at the Academy of Consultation-Liaison Psychiatry Annual Meeting on November 11, 2021.
